# Early Passage Mesenchymal Stem Cells Display Decreased Radiosensitivity and Increased DNA Repair Activity

**DOI:** 10.1002/sctm.15-0394

**Published:** 2017-05-24

**Authors:** Po‐Kuei Wu, Jir‐You Wang, Cheng‐Fong Chen, Kuang‐Yu Chao, Ming‐Chau Chang, Wei‐Ming Chen, Shih‐Chieh Hung

**Affiliations:** ^1^ Institute of Clinical Medicine, School of Medicine National Yang‐Ming University Taipei Taiwan; ^2^ Department of Orthopaedics & Traumatology Taipei Veterans General Hospital Taiwan; ^3^ Therapeutical and Research Center of Musculoskeletal Tumor Taipei Veterans General Hospital Taiwan; ^4^ Institute of Traditional Medicine, School of Medicine National Yang‐Ming University Taipei Taiwan; ^5^ Institute of Biomedical Sciences Academia Sinica Taipei Taiwan; ^6^ Integrative Stem Cell Center, Chinese Medical University Hospital, Graduate Institute of Clinical Medical Science, China Medical University Taichung Taiwan

**Keywords:** Bone marrow mesenchymal stem cells, Radio‐sensitivity, DNA damage response, Cell cycle, PARP‐1, ATM

## Abstract

Cell therapies using human mesenchymal stem cells (MSCs) have received much attention in the past decade. In pursuit of the therapeutic potential of MSCs, cell expansion is required to generate a great number of cells with desired phenotype and functionality. Long‐term expansion in vitro, however, can lead to altered functions. To explore the changes in DNA damage responses (DDR) in MSCs expanded, DDR pathways following irradiation were characterized in early‐ and late‐passage bone marrow MSCs. Seventy‐two hours after irradiation, the percentage of sub‐G1 cells in early‐passage MSCs did not change significantly. Reduced TUNEL staining was observed in early‐passage MSCs compared to late‐passage MSCs 4 h after irradiation. Comet assay also revealed that early‐passage MSCs were more resistant to irradiation or DNA damages induced by genotoxic agents than late‐passage MSCs. ATM phosphorylation and γ‐H2AX and phospho‐p53 increased in early‐passage MSCs while decreased in late‐passage MSCs. Through inhibition by KU55933, DDR pathway in early‐passage MSCs was shown to be ATM‐dependent. Higher levels of poly (ADP‐ribose) polymerase‐1 (PARP‐1) and PAR synthesis were observed in early‐passage MSCs than in late‐passage MSCs. Knockdown of PARP‐1 in early‐passage MSCs resulted in sensitization to irradiation‐induced apoptosis. Overexpression of PARP‐1 in late passage MSCs could render irradiation resistance. Lower activity of DDR in late‐passage MSCs was associated with rapid proteasomal degradation of PARP‐1. In conclusion, early‐passage MSCs are more irradiation‐resistant and have increased DDR activity involving PARP‐1, ATM and their downstream signals. Stem Cells Translational Medicine *2017;6:1504–1514*

**Significance Statement**

This paper demonstrated that early‐passage MSCs are more irradiation‐resistant than that of late‐passage MSCs. The pathways of DNA damage responses following irradiation were characterized. The results indicated that underlying mechanisms associated with increased DNA damage responses in early‐passage MSCs are linked to poly (ADP‐ribose) polymerase‐1 (PARP‐1), ATM and their downstream signals.

## Introduction

Mesenchymal stem cells (MSCs), the nonhematopoietic stromal cells residing in human bone marrow and most of connective tissues, are able to self‐renew and differentiate into mesenchymal cells and nonmesenchymal cells 1. MSCs are known to be genetically stable through culture expansion, and no tumor is induced after transplantation for a long‐term in vivo 2, 3, 4. Further, MSCs can be easily expanded. Therefore, cell‐based therapies using MSCs have received increasing attention in recent years. Cell expansion of MSCs is required to generate a great number of cells with desired phenotype and functionality to drive the potential therapeutic uses. Thus, the therapeutic application of MSCs largely relies on culture expansion. However, there is a lack of data demonstrating the similarities of expanded MSCs to the native MSCs within their niches 5.

Culture‐related modification on expanded MSCs, such as malignant transformation and in vitro aging may cause adverse reactions and unsatisfactory results 6. Studies have reported that long‐term culture of MSCs can induce aging‐related changes, including altered functions, down regulation of genes involved in cell differentiation, a decrease in focal adhesion organization and cytoskeleton turnover, and diminished mitochondria function 6. Long‐term culture in vitro also increases the accumulation of DNA double strand breaks (DSB), the most deleterious form of DNA damage in causing genomic instability 7. Moreover, accumulation of DNA damages during expansion may contribute to the loss of differentiation potential of expanded MSCs 8.

In response to DNA DSB, eukaryotic cells have evolved a DNA damage response (DDR) process to repair the damaged DNA, induce cell death, or entry into senescence 7. DDR involves many reciprocally regulated molecular components. In the late phases of DDR, post‐translational modifications of proteins such as phosphorylation and ubiquitination are particularly prominent in modulating protein‐protein interactions and protein trafficking, activity, and stability 9. Stem cells possess a similar but superior DNA repair machinery 10 in order to maintain longevity. However, this mechanism also results in an accumulation of mutations, thereby inducing tumorigenesis 11. Research has shown that human MSCs are relatively resistant to irradiation (IR)‐induced damages 12, 13, 14, but little is known about whether MSCs respond to IR differently along with in vitro cultivation. Thus, this study aims to better understand the DNA repair capacity in early and late passages of bone marrow MSCs following IR exposure, and to shed light on the underlying mechanisms by which MSCs mediate to respond IR‐induced DNA damages.

## Materials and Methods

### Antibodies and Reagents

For western blotting, anti‐phospho‐ATM [p Ser1981] mouse monoclonal antibody (MoAb) (Novus Biologicals, NB100‐306; Littleton, CO; https://www.novusbio.com/), anti‐total‐ATM rabbit polyclonal antibody (PoAb) (Novus Biologicals, NB100‐104), anti‐phospho‐Histone H2AX (Ser139) rabbit Ab (Cell Signaling, #2577; Danvers, MA; https://www.cellsignal.com/), anti‐RNF8 rabbit PoAb (Genetex, GTX115176; Irvine, CA; http://www.genetex.com/), anti‐p53 Rabbit MoAb (Cell Signaling, #2527), anti‐poly (ADP‐Ribose) Polymerase‐1 **(**PARP) rabbit PoAb (Cell Signaling, #9542), anti phospho‐p53 Ab, (Cell Signaling, #9584), and anti‐PAR mouse MoAb (Enzo, ALX‐804‐220‐R100; Farmingdale, NY; http://www.enzolifesciences.com/), anti‐alpha‐tubulin (Cell Signaling, #2144), anti‐beta‐actin mouse MoAB (Novus Biologicals, NB600‐501), and KU‐55933 (ATM Kinase Inhibitor) (Selleck Chemicals, S1092; Houston TX; http://www.selleckchem.com/) were used. For immunofluorescence, anti‐phospho‐Histone H2AX (Ser139) rabbit Ab (Cell Signaling, #2577) was used. For flow cytometry, Propidium iodide (Sigma‐Aldrich, 25535‐16‐4; St. Louis, MO; http://www.sigmaaldrich.com/) and FITC‐conjugated anti‐mouse IgG (whole molecule) antibody (Sigma‐Aldrich) were used.

### Cell Culture and IR Exposure

This study was performed under Taipei Veterans General Hospital Institutional Review Board‐approved protocols with informed consent obtained from all participants. The MSCs were isolated from iliac crest bone marrow of three donors (aged 30–56 years) as described in the literature 15 (please see Supporting Information Table 1). Data described in this manuscript was generated from MSCs from one of the donors (donor 1). Data of the other two donors were described in Supporting Information figures. Briefly, mononuclear cells were cultured in complete culture medium (CCM) consisting of α‐MEM (Gibco), 16.6% FBS, 100 U/ml penicillin, and 10 µg/ml streptomycin at 37°C with 5% CO_2_ atmosphere. After 1 day, nonadherent cells were discarded by thorough washing the dishes with phosphate‐buffered saline (PBS) and the adherent cells were continuously cultured with medium changes twice per week. At day 14, MSCs were replated at density of 4,000 cells/cm^2^, followed by passage every 7 days when cells reached sub‐confluence. Early‐passage (passage 2∼3) and late‐passage (passage > 10) MSCs were exposed to **γ**‐IR at doses of 4 Gy or 8 Gy using a Maintenance Millennium Sample Irradiator containing a ^137^Cs source at a dose rate of approximately 102 cGy/minute.

### Colony‐Forming Assay After IR Exposure

An assay was performed to evaluate the colony formation ability of early‐passage and late‐passage MSCs after IR exposure (8 Gy). Briefly, aliquots of 1 × 10^4^ cells were seeded in 25‐cm^2^ flasks on the day before IR exposure. After IR exposure (8 Gy), the flasks were incubated at 37°C and 5% CO_2_ for 14 days with medium change twice a week. Cells were then fixed with 10% formalin for 15 minutes, followed by washing using ddH_2_O for twice and stained with 0.1% crystal violet for 30 minutes at room temperature. Cell colony counting was performed on a microscope.

### Flow Cytometry Analysis of Cell Cycle and Detection of Apoptotic Cells

For flow cytometry analysis, early and late passage MSCs at different time points following IR (8 Gy) were resuspended in PBS containing 20 μg/l propidium iodine (PI), 0.1% triton‐X, and 0.2g/l ribonuclease A. Cells were fixed and subjected to flow cytometry analysis by a BDFACSCan to II flow cytometer (BD Biosciences, San Jose, CA). The percentages of the cells in the G0/G1, S, G2, and M phases and the apoptotic cells with degraded DNA in sub‐G1were determined by Flow Jo software (Tree Star, Inc.; Ashland, OR; https://www.flowjo.com/).

### Comet Assay

The Comet assay was carried out by using OxiSelect 96‐well Comet Assay Kit (Cell Biolabs, Inc, STA‐355; San Diego, CA; http://www.cellbiolabs.com/) based on a protocol modified from previously described procedures 16. Briefly, aliquots of 1 × 10^5^ cells per ml were suspended in comet agarose solution at a final concentration of 10% (v/v) and then loaded onto 96‐well comet slide. The slides were immersed in a lysis buffer (2.5 M NaCl, 100 mM EDTA, 1× lysis buffer) at 4°C for 1 hour and change to alkaline solution at 4°C for 30 minutes. Slides were then placed on a horizontal gel electrophoresis unit containing alkaline solution (0.3 M NaOH, 1 mM EDTA) for 15 minutes. Electrophoresis was conducted for the next 15 minutes at 300 mA at an ambient temperature of 4°C. The slides were stained with Vista Green DNA Dye for 15 minutes, and then checked immediately with a microscope. The mean tail moment (percentage of DNA in the tail × tail length) of the individual cells were quantified using CaspLab‐Comet Assay Software, in a blinded manner by counting a minimum of 50 cells per condition in independent experiments.

### Western Blotting

Cell lysates were prepared with “Allele Extract‐M Protein Reagent” lysis buffer (Allele Biotechnology; San Diego, CA; http://www.allelebiotech.com) containing protease and phosphatase inhibitor cocktail (Thermo Fisher Scientific; Waltham MA; https://www.thermofisher.com/). After separating by SDS‐PAGE, proteins were transferred to PVDF membranes and then incubated with optimized antibody dilutions. Chemiluminescence detection was performed by using a UVP BioSpectrum 600 Image System (UVP; Upland, CA; https://www.uvp.com/).

### Immunofluorescence and Immunohistochemical Staining

MSCs were cultured on glass coverslips (Thermo Fisher Scientific) prior to IR. All cultures were fixed in ice‐cold acetone for 10 minutes (Sigma‐Aldrich), permeabilized in 0.5% Triton X‐100 solution (Sigma‐Aldrich) for 15 minutes, and incubated with the indicated primary antibodies at 4°C overnight. Following incubation with the corresponding secondary antibodies at 37°C for 1 hour, cells were mounted in diamidino‐2‐phenylindole (DAPI) solution (Genetex). All images were captured using a Zeiss LSM 700 Laser confocal microscope with Zen 2009 light edition. Quantification of γ‐H2AX foci per cell (total of 50 cells per time point) was randomly selected.

### TUNEL Assay

The TUNEL assay was performed using in In Situ Cell Death Detection Kit, POD (Roche; Basel, Switzerland; http://www.roche.com/) according to the manufacturer's instructions. In brief, cells were fixed in 4% (wt/vol) paraformaldehyde and permeabilized with 0.3% Triton X‐100 (vol/vol). The cells were washed and then incubated with TUNEL reaction mixture at 37°C for 1 hour. The slides were then examined under a Zeiss AXIO Imager A1 microscope. Cell apoptosis rate was determined by counting the number of TUNEL‐positive nuclei and expressed as a percentage of cells counted.

### Generation of Poly (ADP‐Ribose) Polymerase‐1 Knockdown MSC Cells

For PARP‐1 silencing, lentiviral constructs expressing short hairpin RNA (shRNA) targeting human PARP‐1 gene (TRCN0000007932) were purchased from National Science Council RNAi core facility, Academia Sinica, Taiwan. For infection with lentivirus, MSCs, 1 × 10^5^, were seeded in a 6‐cm culture dish 1 day prior to infection and incubated overnight. The next day, old media was replaced with fresh media containing 8 μg/ml of polybrene and the culture dish was incubated for 20 minutes. Infection was conducted at MOI of 3. After incubation for 24 hours, old media was replaced with fresh media containing 5 μg/ml puromycine to select stably transduced cells.

### Detection of mRNA Expressions Using Quantitative RT‐PCR

The total RNA was extracted by using TRIZOL reagent according to the manufacture's instructions (Invitrogen; Carlsbad, CA; https://www.thermofisher.com/). First‐stranded cDNA was synthesized using 5 μg total RNA by RT‐PCR kit (Lucigen; Middleton WI; http://www.lucigen.com/) and analyzed by quantitative PCR SYBR Green (Promega; Madison WI; https://worldwide.promega.com/). The related PCR primers for PARP‐1 were F: 5′ AGTGACAGGCAAAGGCCAGGA 3′ and R: 5′ CGCACCTGGCCCTTTTCTATC 3′, and DNA polymerase was activated by incubating wells at 95°C for 5 minutes, followed by 35 cycles of 0.5 minutes at 94°C, 0.5 minutes at 58°C, and 1 minute at 72°C.

### Overexpression of PARP‐1 in MSCs

For overexpression of PARP‐1, MSCs were transduced with PLAS2w vector carrying full length PARP‐1 gene from National Yang‐Ming University VYM Genome Research Center. In brief, PLAS2w plasmid was digested by SbfI/AgeI restriction enzymes (New England Biolabs; Ipswich MA; https://www.neb.com/) and the full length of PARP‐1 gene was ligased by T7 DNA ligase (New England Biolabs). Viral production of the constructed plasmid with lentivirus system was performed by National Science Council RNAi core facility, Academia Sinica, Taiwan.

### Statistical Analysis

All experiments were performed in triplicate. The differences between more than two groups were compared by one‐way or two‐way ANOVA with Bonferroni post hoc test. Difference of nonparametric data was compared by Wilcoxon signed rank test. A *p* value less than .05 (*p* < .05) was considered as significant and labeled with *, *p* < .05; **, *p* < .01; ***, *p* < .005.

## Results

### Early‐Passages Human MSCs are Less Sensitive to IR Treatment Than Late‐Passage MSCs

**Figure 1:**
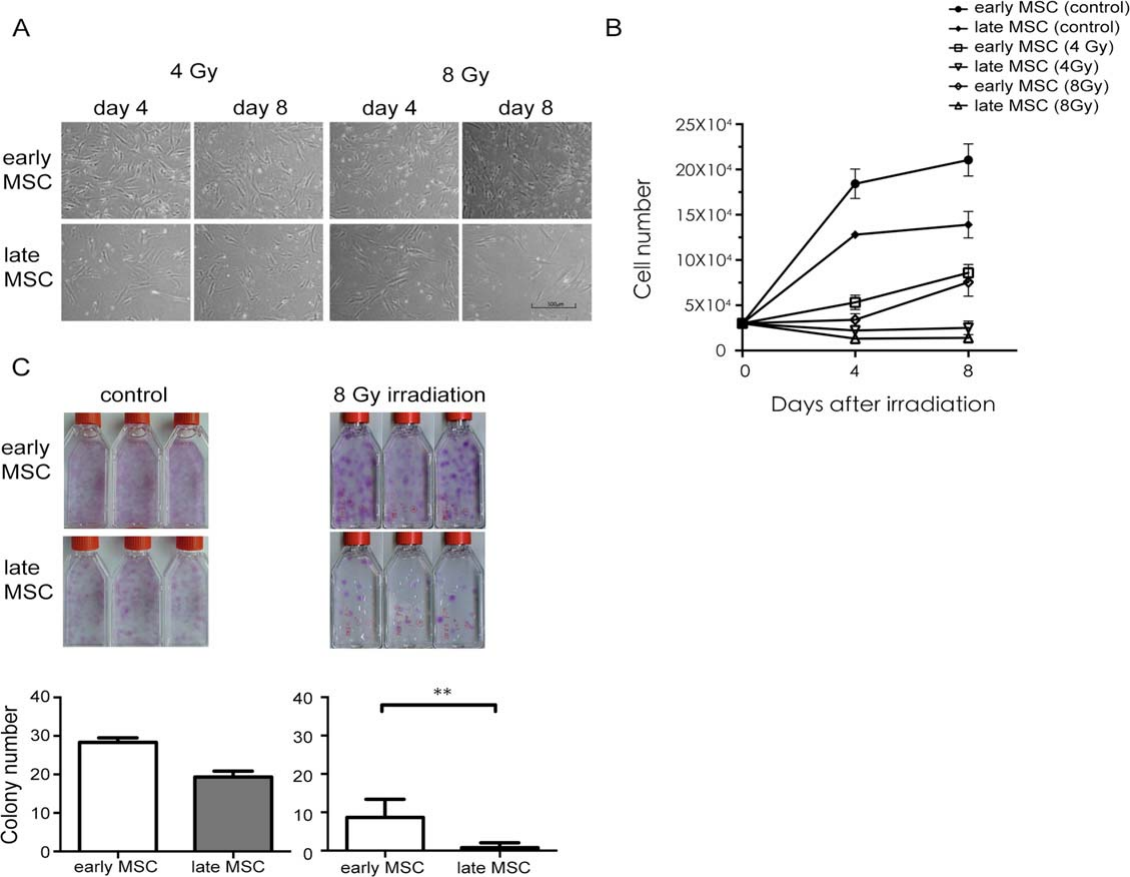
Early‐passage MSCs are to γ‐irradiation treatment than late‐passage MSCs. **(A)**: Cell morphology of early‐ and late‐passage MSCs after γ‐irradiation at 4 Gy and 8 Gy (scale bar, 500 µm). **(B)**: Quantification of early‐ and late‐passage MSCs untreated (control) and treated with γ‐irradiation at 4 Gy and 8 Gy. Cell numbers were determined by Trypan blue exclusion method with the use of an automated cell counter at 0, 4, 8 days postirradiation. Data are presented as mean ± SD of three independent experiments using MSCs from one individual. **(C)**: Quantification of the colony numbers for early‐ and late‐passage MSCs untreated (control) and treated with γ‐irradiation. Aliquots of 1 × 10^4^ early‐ or late‐passage MSCs were seeded into 25‐cm^2^ flasks, followed by irradiation at 8 Gy. Cells were stained with crystal violet (upper panel) and colony numbers were counted (lower panel). Data are presented as mean ± SD of MSCs from three different individuals, each performed in triplicate. **, *p* < .01 (Wilcoxon signed rank test). Abbreviation: MSCs, mesenchymal stem cells.

To determine whether the IR impact varied along with the passage numbers, early‐ (2–3 passages) and late‐passage (>10 passages) MSCs were exposed to irradiation. Changes in cell morphology and numbers were analyzed at various time points after IR. Four or eight days following IR exposure at 4 Gy or 8Gy, early‐passage MSCs showed a normal fibroblastic morphology, maintained a regular size, and were able to increase in number (Fig. 1A, 1B), albeit the growth rate decreased markedly comparing to that of unirradiated MSCs (data not shown). In contrast, a more podia morphology and a cease of proliferation were observed in late‐passage MSCs (Fig. 1A, 1B). Early‐passage MSCs formed significantly more colonies in colony‐forming assay than late‐passage MSCs after 14 day of IR at 8Gy (Fig. 1C, *p* < .01). Together, these results indicate that early‐passage MSCs are less radio‐sensitive comparing to late‐passage MSCs.

### Early‐Passage MSCs are More Resistant to IR‐Induced Apoptosis

**Figure 2:**
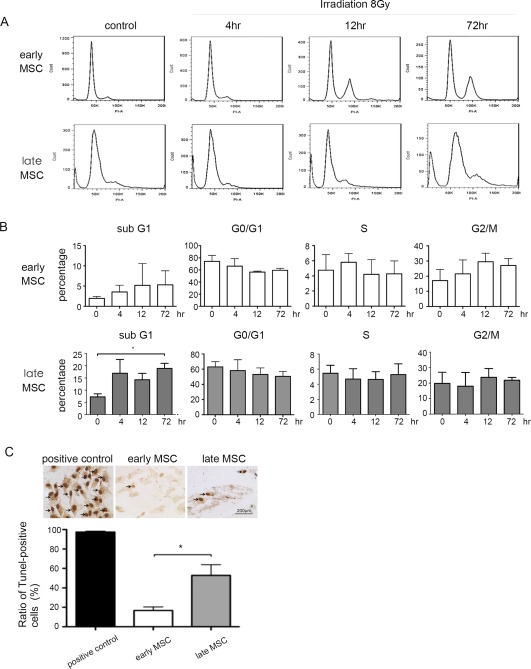
Early‐passage MSCs are more resistant to irradiation‐induced apoptosis than late‐passage MSCs. Cultures of early‐ or late‐passage MSCs were irradiated at 8 Gy. **(A)**: Flow cytometric analysis for cell cycle distribution was performed at indicated time points after irradiation. **(B)**: The graph showing the ratios of MSCs in sub‐G1, G0/G1, S, and G2/M phases. Data are presented as mean ± SD of three independent experiments using MSCs from two individuals (duplicate in one individual and one in the other individual). *p* >.05 by Wilcoxon signed rank test. **(C)**: upper panel: TUNEL staining for analyzing apoptotic cells at 4 h of 8 Gy (magnification: 400×). (C): lower panel: Significant difference was observed in the percentages of TUNEL‐positive cells. Data are presented as mean ± SD of three independent experiments using MSCs from one individual. *, *p* < .05 (Wilcoxon signed rank test). Abbreviation: MSCs, mesenchymal stem cells.

To study the features of irradiated MSCs, flow cytometric analyses were performed to identify differences in apoptotic and proliferative responses to IR exposure at 8 Gy (Fig. 2A). The percentage of sub‐G1 cells in late‐passage MSCs was increased significantly by IR after 72 hours, while the percentage of sub‐G1 cells in early‐passage MSCs essentially remained the same. Although the percentage of early‐passage MSCs in G0/G1 phase was higher than late‐passage MSCs before IR exposure, the G0/G1 populations of both MSCs decreased after IR exposure, but the difference between early and late passage MSCs did not reach to statistical significance (Fig. 2B). IR (8 Gy) did not result in noticeable changes in the population of S phase cells over 72 hours post‐IR exposure. Although the fraction of early‐passage MSCs in G0/G1 phase declined in early‐passage MSCs, an opposite trend was observed in G2/M phase cells. In addition, no difference was observed in the percentages of G2/M cells between the two MSCs prior to IR exposure. Early‐passage MSCs started to substantially accumulate in G2/M phase within 4 hours after IR exposure, peaked at 12 hours and plateaued up to 72 hours. However, the late‐passage MSCs showed no increased arrest in G2/M phase despite having minor differences in distributions (Fig. 2B). TUNEL assay also revealed that only a few apoptotic cells were detected in the early‐passage MSC at 4 hours after IR exposure (Fig. 2C). In contrast, TUNEL‐positive cells with shrunken cell bodies and condensed nuclei were significantly increased in the irradiated late‐passage MSCs (16.7% ± 3.7% vs. 52.9% ± 11.1%, *p* < .05, Fig. 2C). These results together suggest that early‐passage MSCs are more resistant to IR‐induced apoptosis and have a more profound and transient G2/M arrest after IR treatment.

### Early Passage MSCs are Less Sensitive to DNA Damaging Agents

**Figure 3:**
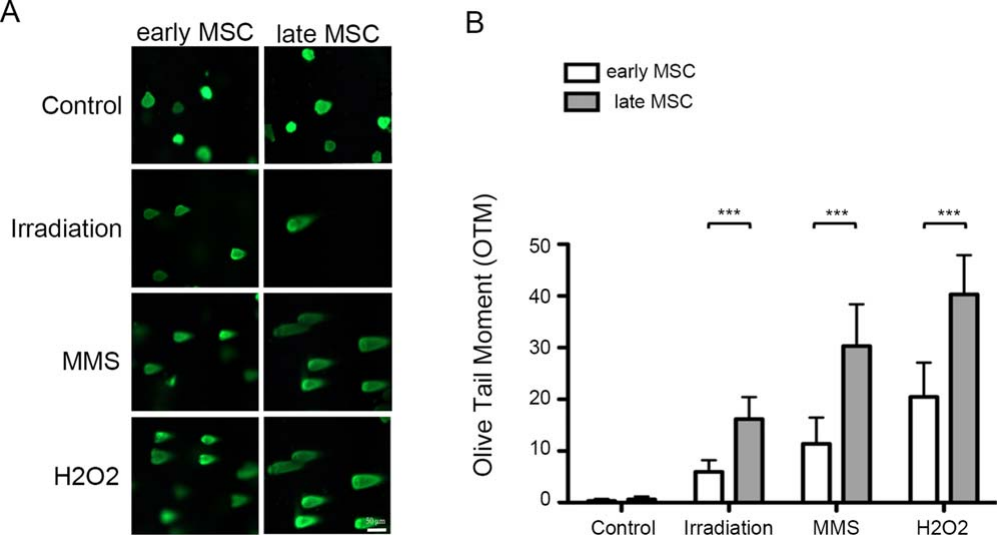
Early‐passage MSCs are more resistant to γ‐irradiation‐ and genotoxic agents‐induced DNA damage than late‐passage MSCs. **(A)**: Cultures of early‐ and late‐passage MSCs without (control) and with subjection to 8 Gy irradiation (4 hours), 10 mM MMS (1 hour), and 50 μM H_2_O_2_ (30 minutes) were measured in olive tail moment for the extent of DNA damage (magnification: 200×). **(B)**: Cells were quantified in comets core and presented as the percentage of DNA in the tail (DNA% × tail moment length). Data are presented as mean ± SD of three independent experiments using MSCs from one individual. ***, *p* < .001 (Wilcoxon signed rank test). Abbreviations: MMS, methyl methanesulfonate; MSCs, mesenchymal stem cells.

As the evidence from above suggested that the apoptosis of MSCs reflects their functional response to IR‐induced DNA damage, comet assay was performed to assess the extent of DNA damage in both cells. Given that methyl methanesulfonate (MMS) and H_2_O_2_ are well known to cause DNA DSB and have been commonly used as comparative genotoxic agents in determining DNA damage 17, 18, we compared the extent of DNA DSB damage between early‐ and late‐passage MSCs after treatment with MMS, H_2_O_2_, and 8 Gy of IR by comet assay. Comparing to control cells that showed almost no DNA damage, MSCs exposed to these insults exhibited “comet tails” (Fig. 3, left). However, the average tail length in early‐passage MSCs was significantly shorter than that of late‐passage MSCs in all tested agents (Fig. 3, right; *p* < .001). These observations suggest that early‐passage MSCs are more resistant to DNA damage in the presence of genotoxic agents.

### More Efficient Repair of DNA DSB in Early‐Passage MSCs

**Figure 4:**
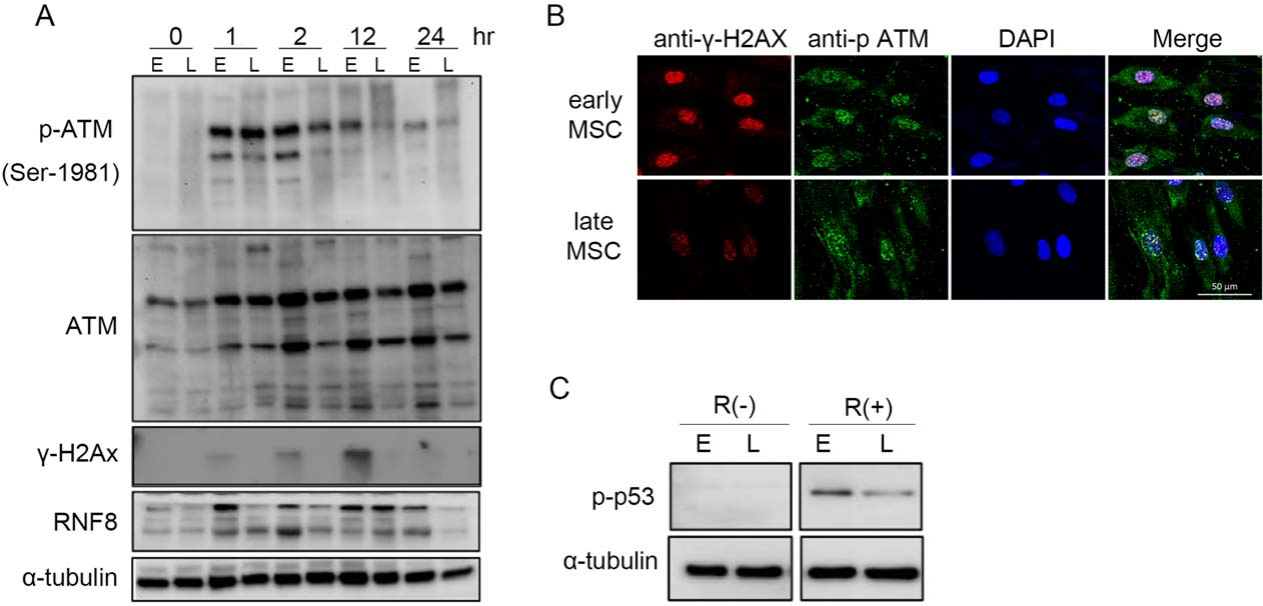
Response of MSCs to DNA damage. **(A)**: Cultures of early‐ and late‐passage MSCs were subjected to un‐treated (0 hour) and treated with γ‐irradiation at 8 Gy irradiation, followed by western blot analysis at indicated time points. **(B**, **C)**: Cultures of early‐ and late‐passage MSCs before or 2 hours after 8 Gy irradiation were subjected to immune‐fluorescence (B) (magnification: 400×) and western blot analysis (C). α‐tubulin is shown as a loading control. The results are representative of three individual experiments. Data are presented as mean ± SD of three independent experiments. Abbreviation: MSCs, mesenchymal stem cells.

To look into the potential DNA DSB repairing capacity and to identify the DDR pathways of early‐ and late‐passage MSCs, several key DDR components were analyzed, including phosphorylated‐ataxia telangiectasia mutated (p‐ATM), histone variant γ‐H2AX (phosphorylated at Ser 139), and RNF8 (Fig. 4). ATM phosphorylation was evident in early‐passage MSCs at 1 hour, peaked at 2 hours, and plateaued for at least 24 hours after 8 Gy of IR exposure. The p‐ATM levels in late‐passage MSCs elevated immediately 1 hour after IR exposure and diminished quickly 2 hours after IR (Fig. 4A). The results show that higher levels of ATM and p‐ATM in early‐passage cells. Gradually increased γ‐H2AX (phosphorylated form) level was detected at 1 hour and peaked at 12 hours after exposure to 8 Gy of IR in early‐passage MSCs, and nearly returned to control levels 24 hours later; however, the γ‐H2AX level in late‐passage MSCs was almost unchanged comparing to basic level before IR. The recruited downstream repair factor, RNF8, was also elevated within 1 hour and increased dramatically at 12 hours post IR in early‐passage MSCs, but this feature of RNF8 up‐regulation did not appear in late‐passage cells.

Consistently, increased numbers of γ‐H2AX and p‐ATM foci colocalized in the nuclei were detected at 2 hours after 8 Gy of IR treatment, indicating that DNA DSB repair was more prominent in early‐passage MSCs (Fig. 4B). In addition, the level of phosphorylated p53 (p‐p53), another established checkpoint protein driven by ATM 19, was also increased more significantly at 2 hours after 8 Gy of IR treatment in the early passage MSCs (Fig. 4C). Similar findings were observed in the MSCs isolated from another two individuals (Supporting Information Fig. 2). These data suggest that early‐passage MSCs can increase the activation of several DDR components and DNA DSB repairing capacity after IR compared to late‐passage MSCs.

### DNA DSB Repair in Early Passage MSCs is ATM‐Dependent

**Figure 5:**
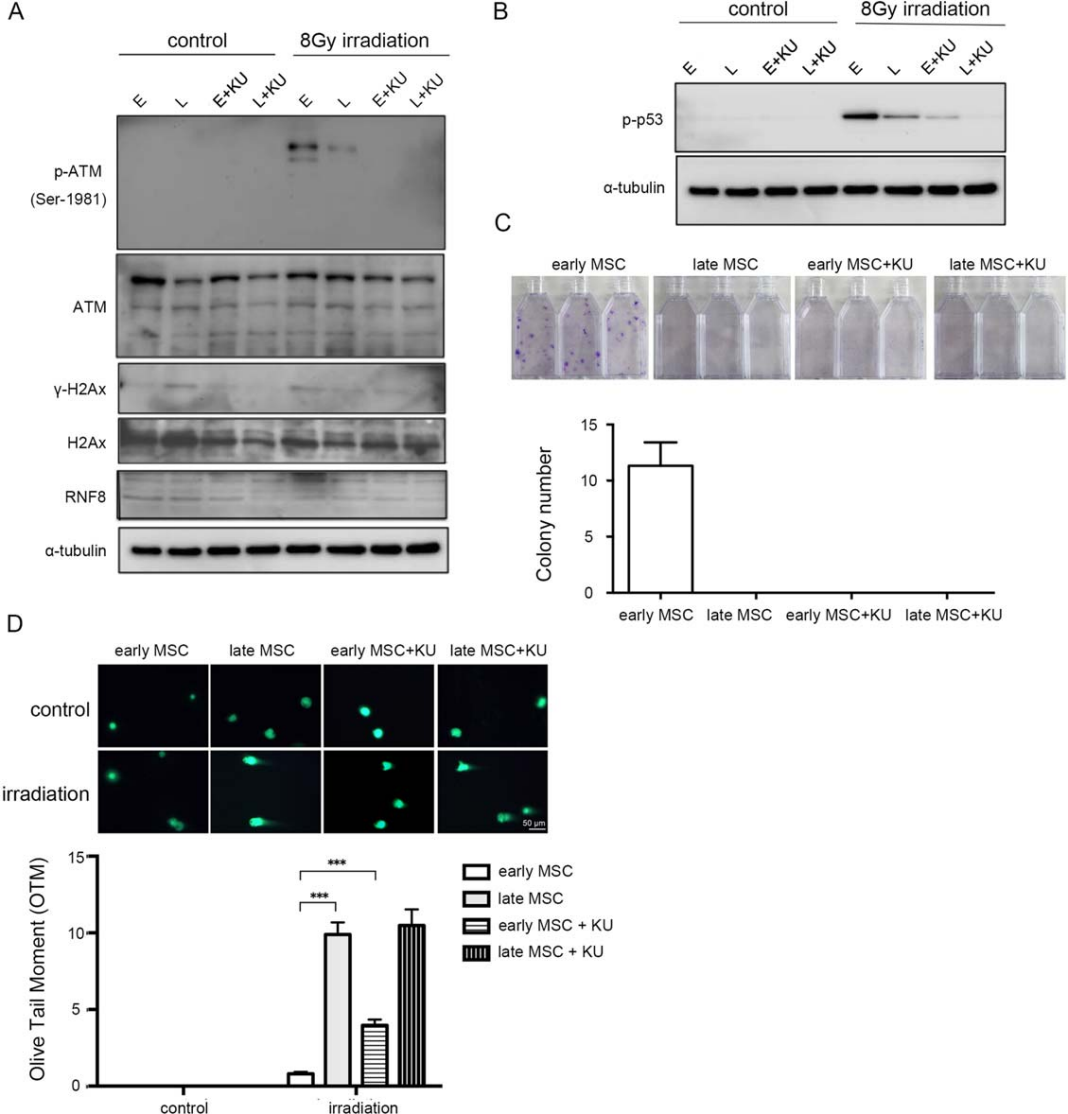
Increased DNA double strand break repair in early‐passage MSCs is ATM‐dependent. Early‐ and late‐passage MSCs without or with 2‐hour KU55933 pretreatment (10 µM) were subjected to untreated (control) and treated with γ‐irradiation at 8 Gy, followed by western blot analysis 2 hours later **(A**, **B)**, colony formation assay 14 days later **(C)**, measurement (**D** upper panel) and quantification of DNA damage **(**D lower panel**)** in olive tail moment (OTM) as the percentage of DNA in the tail (DNA% × tail moment length) 12 hours later (magnification: 200×). The results are representative of three independent experiments using MSCs from one individual. Data are presented as mean ± SD. ***, *p* < .001 (Wilcoxon signed rank test). Abbreviation: MSCs, mesenchymal stem cells.

We next examined whether the stronger capacity of DNA DSB repair was ATM‐dependent in early‐passage MSCs. A highly reversible ATM inhibitor, KU55933 20, was used to treat early‐ and late‐passage MSCs for 2 hours before exposure to IR. The results showed that ATM protein levels were higher in early‐passage MSCs with or without KU55933 treatment when compared with corresponding late‐passage cells. As expected, the IR‐induced increase in p‐ATM level in early‐passage MSCs was inhibited by KU55933 (Fig. 5A). Consequently, the levels of downstream DNA repair factors, including γ‐H2AX, RNF8, and p‐p53 21, in response to IR were also reduced upon ATM inhibition (Fig. 5B). Colony formation assay (Fig. 5C) showed that no colony formed in early‐passage MSCs treated with KU55933, and late‐passage MSCs treated or untreated with KU55933. In addition, MSCs exposed to KU55933 and IR exhibited significant increase in comet tails. Although the difference in DNA damage was significant for early‐passage MSCs, the extent of DNA damage was much higher in late‐passage cells (Fig. 5D). These results suggest that DNA DSB repair is ATM‐dependent in both early‐ and late‐passage MSCs, but the DNA DSB repairing capacity is reduced in late‐passage cells.

### PARP‐1 Protein is Essential for the Accumulation of p‐ATM in IR Treated MSCs

**Figure 6:**
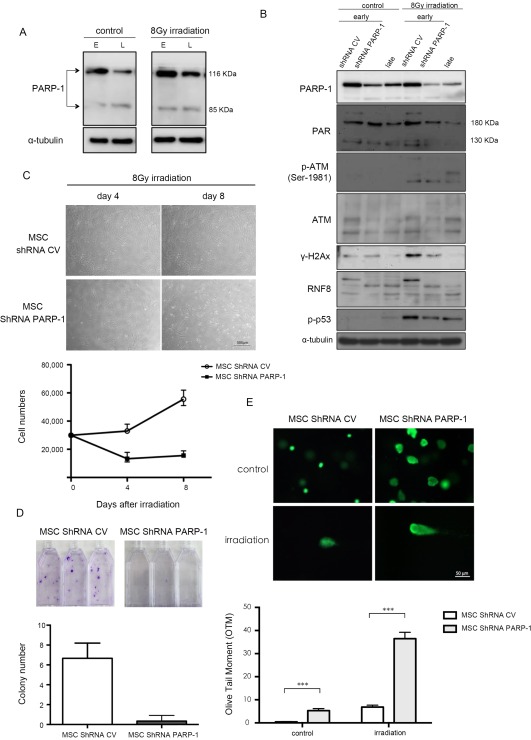
Increased PARP‐1 expression in early‐passage MSCs is required for the DNA double strand break repair. **(A)**: Early‐ and late‐passage MSCs before (control) or 2 hours after 8 Gy irradiation were subjected to western blot analysis. **(B–E)**: MSCs lentivirally transduced with control (shRNA CV) or PARP‐1 specific shRNAs (shRNA PARP‐1) without or with 8 Gy irradiation were subjected to western blot analysis 2 hours later (B), cell number counting at indicated time points (C) (magnification: 100×), colony formation assay 14 days later (D). Measurement **(**E left panel**)** and quantification of DNA damage **(**E right panel**)** in olive tail moment (OTM) as the percentage of DNA in the tail (DNA% × tail moment length) were performed 12 hours later (magnification: 200X). Data are presented as mean ± SD of three independent experiments using MSCs from one individual. ***, *p* < .001. Abbreviations: MSCs, mesenchymal stem cells; PARP‐1, poly (ADP‐Ribose) polymerase‐1.

PARP‐1 is activated in response to DNA damage through a complex signal mechanism dependent upon the damage response kinase ATM 22. Poly(ADP‐ribosyl)ation is not only associated with sensing DNA damage in the process, but is also involved in repairing protein accumulation. To evaluate the link between ATM kinase and PARP‐1 in early‐ and late‐passage MSCs, PARP‐1 levels were measured by immunoblotting analyses (Fig. 6A). Results indicated that the PARP‐1 protein level was higher in early‐passage MSCs prior to IR. The PARP‐1 levels of both early‐ and late‐passage MSCs increased in response to 8 Gy of IR exposures, and the PARP‐1 level of early passage MSCs increased more than that of late passage MSCs. PARP‐1 involvement in MSC DDR response was demonstrated by comparing lentiviral‐mediated RNAi of PARP‐1. PARP‐1 knockdown led to reduced PAR synthesis in early‐passage MSCs. The IR‐induced increase of p‐ATM level was diminished in early‐passage MSCs after PARP‐1 knockdown. Decreased phosphorylation of ATM substrate H2AX (γ‐H2AX) was also detected (Fig. 6B). PARP‐1 knockdown led to decreased cell viability following IR in early‐passage MSCs and induced cell‐proliferation arrest at 4 days after 8 Gy IR (Fig. 6C). The colony formation ability was also reduced in MSCs with PARP‐1 knockdown than that transduced with control vector but the decrease is not statistically significant (Fig. 6D). The comet assay showed a significantly longer comet tail intensity in irradiated early MSCs with PARP‐1 knockdown than that without PARP‐1 knockdown (control vector) (Fig. 6E).

### More Rapid Degradation of PARP‐1 Protein in Late‐Passage MSCs

To investigate the mechanism mediating PARP‐1 protein levels in early‐ and late‐passage MSCs, we first showed that there is no difference in mRNA levels between early‐ and late‐passage MSCs (Supporting Information Fig. 3A). We then analyzed PARP‐1 protein levels in the presence or absence of proteasome inhibitor MG132. After cells were treated with protein synthase inhibitor cycloheximide (CHX) for various lengths of time, PARP‐1 maintained at the same level in early‐passage MSCs with and without MG132 treatment (Supporting Information Fig. 3B). However, in late‐passage MSCs, PARP‐1 rapidly degraded in the absence of MG132 and maintained the same in the presence of MG132 (Supporting Information Fig. 3B). These results indicate that PARP‐1 protein rapidly degraded through the participation of proteasomal pathway in late‐passage MSCs. However, the protein levels of the putative PPAR ligase, such as checkpoint with fork‐head associated and ring finger (CHFR) 23, were not different between early‐ and late‐passage MSCs (data not shown).

### Overexpression of PARP‐1 Is Associated with Increase in DNA Damage Responses, Survival and DSB Repair

**Figure 7:**
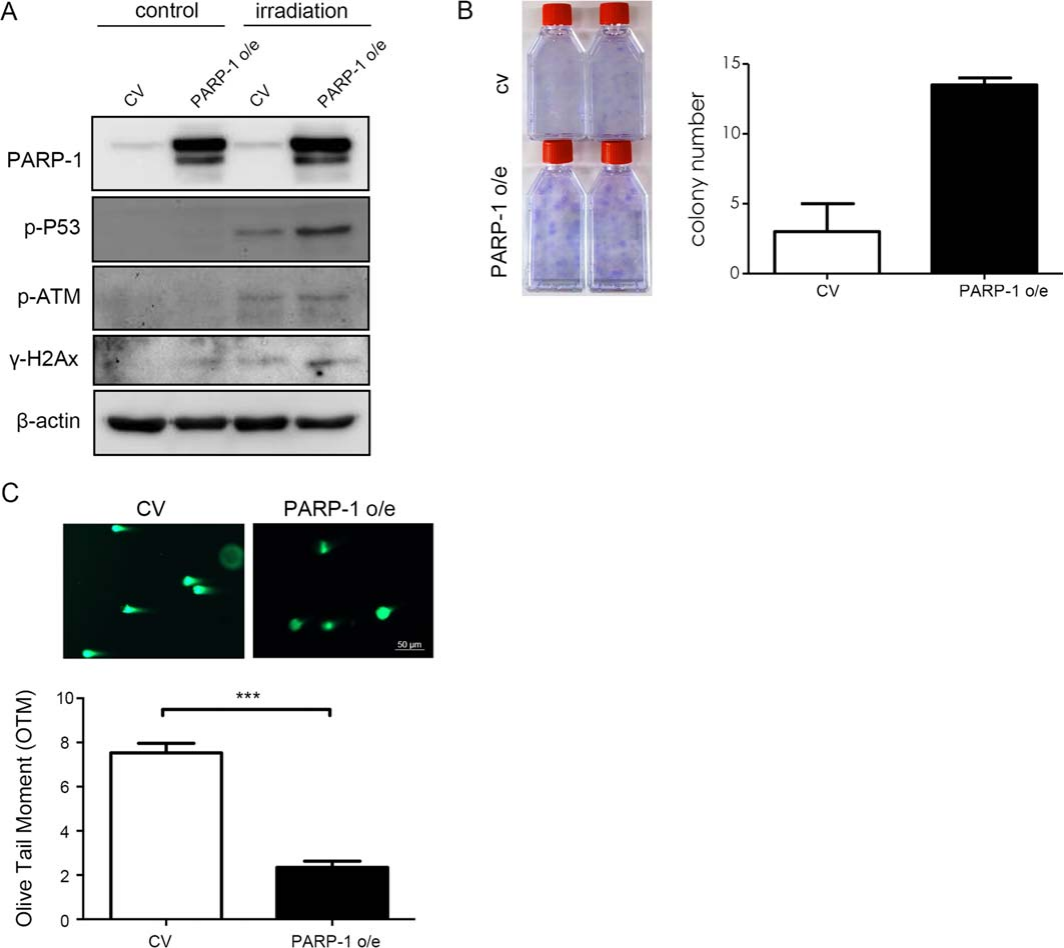
Overexpression of PARP‐1 in late‐passage MSCs increases DNA damage responses, survival and DNA double strand break repair. Late‐passage MSCs lentivirally transduced with control (CV) or PARP‐1 (PARP‐1) without or with 8 Gy irradiation were subjected to western blot analysis 2 hours later **(A)**, colony formation assay 14 days later **(B)**, and measurement (**C** upper panel) and quantification of DNA damage (C lower panel) in olive tail moment (OTM) as the percentage of DNA in the tail (DNA% × tail moment length) 12 hours later (magnification: 200×). Data are presented as mean ± SD of three independent experiments using MSCs from one individual. ***, *p* < .001 (Wilcoxon signed rank test). Abbreviations: MSCs, mesenchymal stem cells; PARP‐1, poly (ADP‐Ribose) polymerase‐1.

To demonstrate PARP‐1 restoration in late‐passage MSCs de novo induced radio‐resistance, we first showed that overexpression of PARP‐1 increased the levels of p‐P53, p‐ATM, and γH2AX after IR for 4 hours in comparison to MSCs transduced with control vectors (Fig. 7A). Additionally, overexpression of PARP‐1 promoted colony formation ability of MSCs (Fig. 7B) and accelerated the reduction of DNA damage, as evidenced by decreased comet tail intensities (Fig. 7C). These results indicated that PARP‐1 was required for p‐ATM accumulation and consequent phosphorylation of H2AX in IR treated MSCs. Taking together, these findings suggest that PARP‐1 is involved in facilitating recruitment of DNA DSB repair factors in MSCs following IR exposure, and the extent is higher in early‐passage cells.

## Discussion

We have demonstrated that, despite IR‐induced cell cycle arrest and suppression of proliferative activity, early‐passage MSCs are substantially more radio‐resistant than late‐passage MSCs. Significant apoptotic and necrotic changes were not observed in early‐passage MSCs after treated with IR at doses of 4 and 8 Gy, while late‐passage MSCs showed profound increases in radio‐sensitivity associated with apparent DNA damage and apoptosis after IR exposure. Similarly, early‐passage MSCs were also more resistant to genotoxic agents and exhibited higher DNA DSB repairing capacities. Consistent with previous studies in MSCs 13, 14 and other stem cells, such as haematopoietic stem cells 24, 25 and embryonic stem cells 26, 27, our findings provide the evidence supporting that IR activates key DNA damage responses and cell cycle checkpoints in MSCs, especially at early passages.

In this study, the hypothesis of PARP‐1‐dependent DNA damage response was verified by demonstrating the involvement of PARP‐1 activity in DNA damage‐induced signaling cascade in MSCs. Elevated level of PARP‐1 in early‐passage MSCs also was associated with radio‐resistant capacity and upregulated levels of p‐P53, p‐ATM, and γH2AX. We also showed that the lower PARP‐1 level in late‐passage MSCs was a result of more rapid degradation of PARP‐1 through proteasomal pathway. However, no significant difference was found in the levels of CHFR that was known to degrade PARP‐1. Further studies are required to clarify the mechanisms of PARP‐1 degradation in MSCs.

Phosphorylation of H2AX on serine 139 (**γ**H2AX) is an indirect marker of DNA strand breaks and replication arrest 28. We found that **γ**H2AX peaked at 12 hours following IR exposure in early‐passage MSCs, but decreased rapidly 24 hours after IR treatment. This was inconsistent with previous study indicating that **γ**H2AX was sustained for up to 48 hours following exposure 13. Other than that, the observation of damage‐induced **γ**H2AX in MSCs was generally consistent with previous reports 13, 29. Decreased **γ**H2AX staining at later times following IR exposure was also in line with DNA damage and repairing mechanism 13, 30. After high dose of IR, phosphorylation of p53 leads to p53 stabilization and downstream induction of p21 expression, thereby maintaining G1 phase arrest 12, 13, 31. Increased p53 level following exposure to IR has been reported in MSCs 13, 32, 33 as well as HSCs 11, 34. However, the resistance of MSCs to IR in the present study was distinct from the reported sensitivity of HSCs to DNA damage induced apoptosis or cell death 11, 34, 35, 36. Thus, in the bone marrow, stem cells of the mesenchymal and hematopoietic lineages may respond differently to the DNA cytotoxic agents.

Following exposure to 8 Gy of IR, there was no observed change in the proportion of MSCs in S phase for up to 72 hours, which was different from a previous study where MSCs decreased in S phase percentage following exposure to 10 Gy of IR 13. These MSCs also increased in the G2/M phase following exposure to 10 Gy of IR 13. This was consistent with our findings in early‐passage MSCs, but not in late‐passage cells. Further experiments are required to understand whether the discrepancy between these studies was driven by differences in irradiation dosages or passage numbers. Furthermore, IR exposure resulted in an observable decreasing trend in arresting both early‐ and late‐passage MSCs in the G0/G1 phase up to 72 hours post IR, and a substantial accumulation of early‐passage MSCs in the G2/M phase. These results indicate that early‐passage MSCs possess more effective cell cycle checkpoints in G2/M following IR exposure than late‐passage MSCs. As homologous recombination (HR) mainly takes place in the G2/M phase and early‐passage MSCs increase in G2/M arrest following IR, it is likely that DNA damage is repaired through error‐free HR in early‐passage MSCs. Given that more late‐passage MSCs were in the G0/G1 phase, the error‐prone nonhomologous end joining (NHEJ) can be the major DNA repair mechanism for late‐passage MSCs 37, which may result in more genomic alterations.

A recent study reported that the hematopoiesis‐supporting activity of MSCs declined after longer time in culture, despite the phenotype and differentiation potentials of MSC preparations were stable up to passage 10 [38]. This study also suggests that phenotype analysis dose not accurately reflect the biological properties of MSCs, and MSCs should be used before too many passages 38. Although the optimal passage number to preserve the IR tolerance of MSCs still needs to be established, this study demonstrated that early‐passage MSCs (passages 2–3) exhibited a significantly higher DDR capacity than late‐passage cells.

## Conclusion

In summary, our data provide evidence supporting the application of MSCs for therapeutic purposes and the passage numbers should be taken into consideration when designing therapeutic strategy.

## Author Contributions

P‐K.W.: conception and design, collection and assembly of data, data analysis and interpretation, manuscript writing; J‐Y.W.: collection and assembly of data, data analysis and interpretation, administrative support; W‐M.C.: financial support, conception and design, data analysis and interpretation; S‐C.H.: provision of study material, conception and design, analysis and interpretation, scientific discussions, manuscript writing, and final approval of manuscript.

## Disclosure of Potential Conflicts of Interest

The authors indicated no potential conflicts of interest.

## Supporting information

Supporting Information

Supporting Information

Supporting Information

Supporting Information

Supporting Information
