# Supplementary figures and images for: Early Passage Mesenchymal Stem Cells Display Decreased Radiosensitivity and Increased DNA Repair Activity
Source: Stem Cells Transl Med. 2017 May 24;6(6):1504–14. doi: 10.1002/sctm.15-0394 (PMC5689774; doi:10.1002/sctm.15-0394)

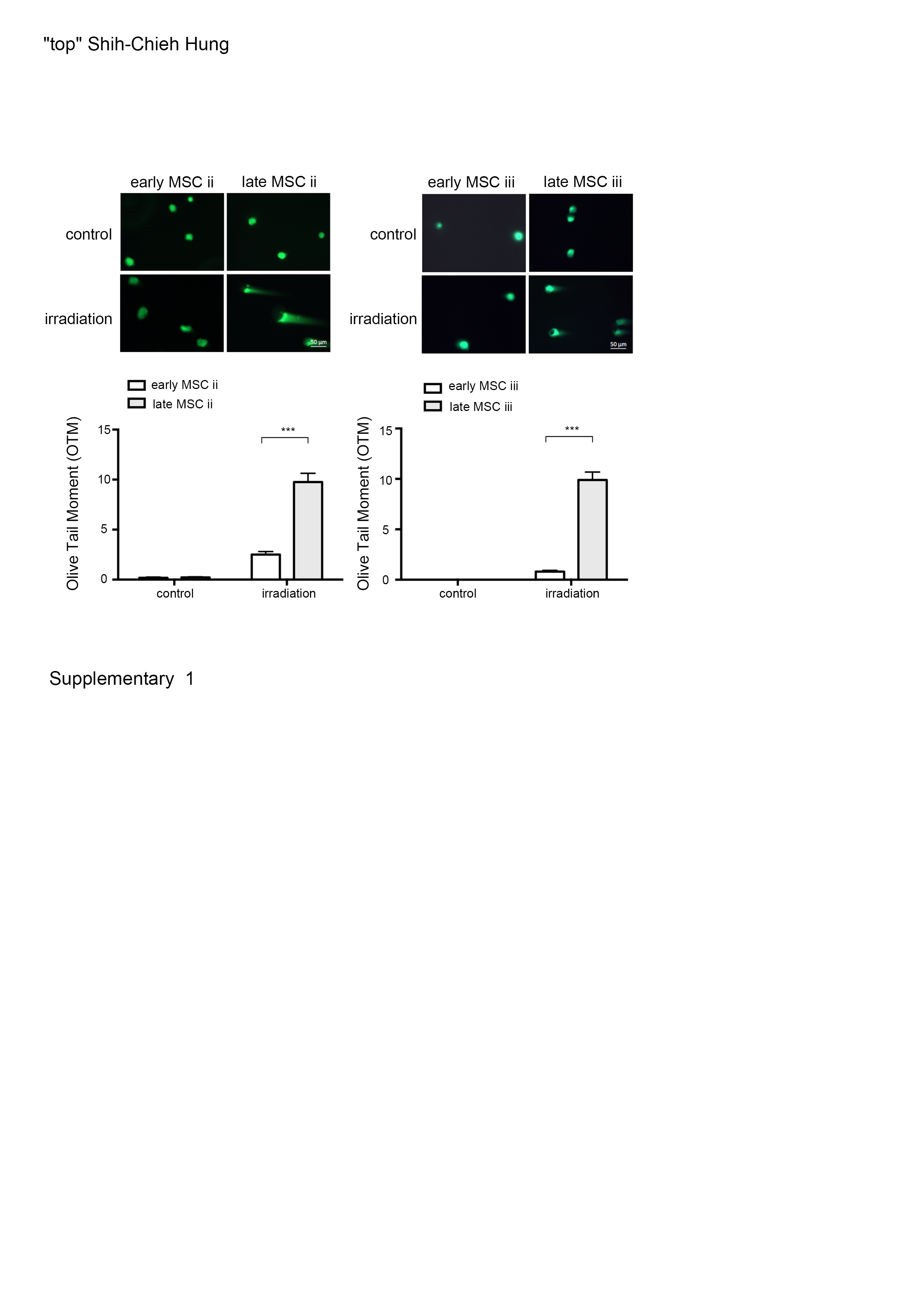

Supplement: Supplementary file 1 — Supporting Information [file SCT3-6-1504-s001.jpg]

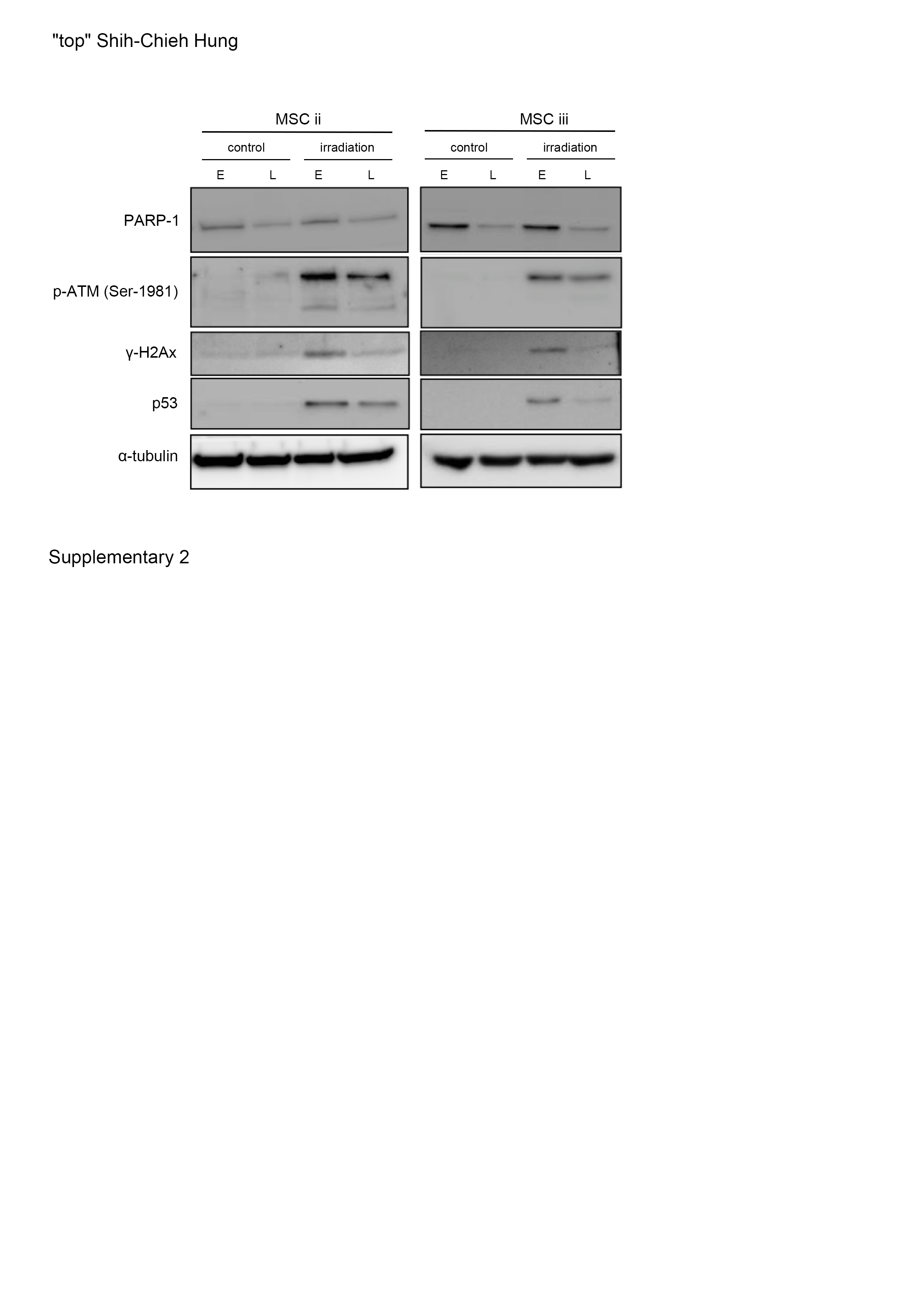

Supplement: Supplementary file 2 — Supporting Information [file SCT3-6-1504-s002.jpg]

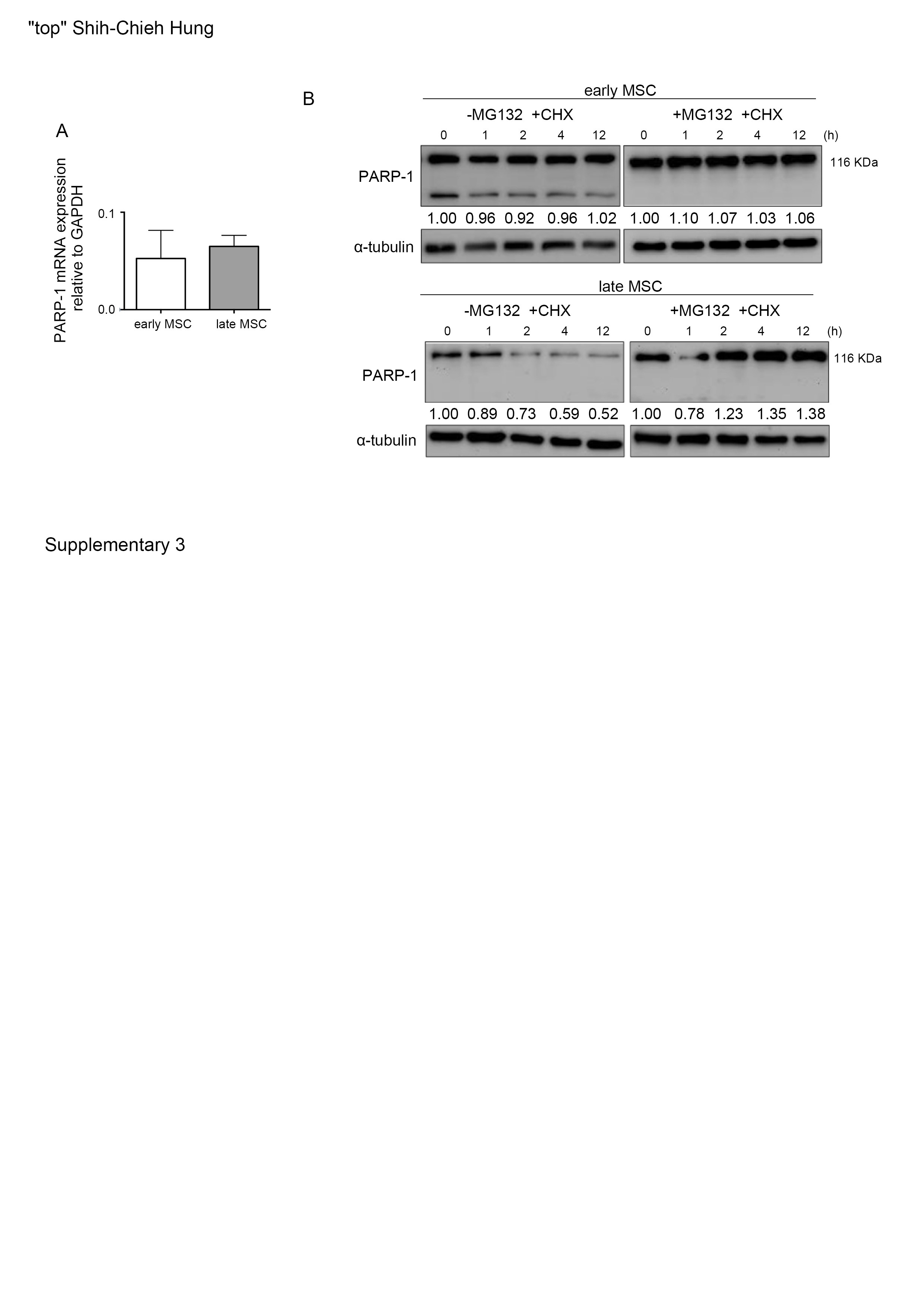

Supplement: Supplementary file 3 — Supporting Information [file SCT3-6-1504-s003.jpg]
