# Supplementary material for: Early Passage Mesenchymal Stem Cells Display Decreased Radiosensitivity and Increased DNA Repair Activity
Source: Stem Cells Transl Med. 2017 May 24;6(6):1504–14. doi: 10.1002/sctm.15-0394 (PMC5689774; doi:10.1002/sctm.15-0394)
Supplement: Supplementary file 4 — Supporting Information [file SCT3-6-1504-s004.docx]

**Supplemental Figure Legends – Hung and Chen**

**Supplemental Figure 1.** Early-passage MSCs isolated from another two individuals have greater DNA double strand break repair than late-passage MSCs.

**(Upper panel)** Cultures of early- and late-passage MSCs without (control) and with irradiation at 8 Gy (4 h) were measured in olive tail moment for the extent of DNA damage. **(Lower panel)** Cells were quantified in comets core and presented as the percentage of DNA in the tail (DNA% × tail moment length) (magnification: 200X). Data are presented as mean ± SD of three independent experiments using MSCs from two different individuals, each performed in triplicate. ****P*<0.001 (Wilcoxon signed rank test).

**Supplemental Figure 2.** Early-passage MSCs isolated from another two individuals increase in DNA damage responses.

Cultures of early- and late-passage MSCs before 8 Gy irradiation were subjected to western blot analysis. α-tubulin is shown as a loading control.

**Supplemental Figure 3.** PARP-1 is rapidly degraded in late-passage MSCs.

**(A):** Quantitative RT-PCR for analyzing the PARP-1 mRNA levels. Data are presented as mean ± SD of three independent experiments using MSCs from one individual. **(B):** Early- and late-passage MSCs were treated without or with MG132 (proteasome inhibitor) in the presence of cycloheximide (CHX, protein synthesis inhibitor) for indicated time points, followed by western blots analysis. α-tubulin was used as a loading control.
