# Supplementary material for: Early Passage Mesenchymal Stem Cells Display Decreased Radiosensitivity and Increased DNA Repair Activity
Source: Stem Cells Transl Med. 2017 May 24;6(6):1504–14. doi: 10.1002/sctm.15-0394 (PMC5689774; doi:10.1002/sctm.15-0394)
Supplement: Supplementary file 5 — Supporting Information [file SCT3-6-1504-s005.docx]

Supplementary Table 1. Information on donor profile

|  | Age | Gender | Race | Health | Harvest site |
| --- | --- | --- | --- | --- | --- |
| Donor 1 | 30 | male | Asian | health | bone marrow |
| Donor 2 (MSC ii) | 56 | female | Asian | health | bone marrow |
| Donor 3 (MSC iii) | 32 | male | Caucasian | health | bone marrow |
